# Antiulcer and Anti-inflammatory Activity of Aerial Parts *Enicostemma littorale* Blume

**DOI:** 10.4103/0975-1483.71629

**Published:** 2010

**Authors:** SP Roy, CM Niranjan, TM Jyothi, MM Shankrayya, KM Vishawanath, K Prabhu, VA Gouda, RS Setty

**Affiliations:** *Department of Pharmacology, S. C. S. College of Pharmacy, Harapanahalli - 583 131, Karnataka, India*; 1 *College of Clinical Pharmacy, King Faisal University, Al Ahsa, 31982, Kingdom of Saudi Arabia*

**Keywords:** Anti-inflammatory, antioxidants, antiulcer, *Enicostemma littorale*

## Abstract

The antiulcer and *in vitro* anti-inflammatory activities of the aerial parts of *Enicostemma littorale* against aspirin, ethanol, and pyloric ligation-induced ulcers in rats and bovine serum albumin denaturation were studied. The extract (200 mg/kg and 400 mg/kg po) was administered to the overnight fasted rats, one hour prior to aspirin / alcohol / pyloric ligation challenge. The ulcer index, tissue GSH levels, and lipid peroxidation levels were estimated in all the models of ulcers and the volume of gastric secretion, acidity, and pH, were estimated in the pyloric ligation model of ulcers. Pretreatment with the extract showed a dose-dependent decrease in the ulcer index (Against Aspirin, ethanol challenge, and pyloric ligation. The prior administration of the extract also reduced the total acidity, free acidity, and volume of gastric secretion, and elevated the gastric pH. In addition, it was also observed that the extract inhibited the serum albumin denaturation in a dose-dependent manner. It may be concluded that the methanolic extract possesses antiulcer activity, and the anti-inflammatory activity of the extract may be attributed to the antioxidant potential, as reported earlier.

## INTRODUCTION

Various mechanisms have been suggested for explaining the pathogenesis of peptic ulcer, which results due to an imbalance between the protective mechanisms and aggressive factors such as pepsin and acid.[1] In addition, the free radicals involved in tissue damage have been reported to play a role in the causation of gastric ulcers. Several classes of drugs are being adopted in the treatment of ulcers to restore the balance between aggressive and protective factors involved in the causation of ulcers. However, administration of drugs for a prolonged period is required to treat ulcers. Prolonged usage of such synthetic agents may cause side effects / drug interactions with concomitantly used drugs or food. It is unaffordable for common man to take such drugs for a prolonged period due to their escalating costs. Therefore, a large section of the world’s population relies on traditional remedies / alternative systems of medicines to treat a plethora of diseases including gastric ulcers.[2]

Hence, in our search for herbal remedies for diseases that require chronic treatment, we found that *Enicostemma littorale* Blume a glorious perennial herb belonging to the family Gentianceae. Upon literature survey it was found that the hot extract from it is being used by tribal healers of interior Gujarat, for the treatment of diabetes, fever, stomach ache, dyspepsia, and malaria. It is also reported to possess antitumor,[3] antiarthritic,[4] hypoglycemic,[5] and antimalarial activities.[6] There are reports that the plant possesses flavonoids, xanthines, and so on, in the aerial parts of this plant.[7] The flavonoids are known to have antioxidant, antiulcer, and anti-inflammatory properties. However, there are no reports on the gastroprotective activity of the plant. As, this plant is reported to contain flavonoids and other related compounds, the methanolic extract of this plant is investigated for gastroprotective and *in vitro* anti-inflammatory activities by using various experimental models of ulcers namely pyloric ligation, ethanol, and aspirin-induced ulcers in albino rats.

## MATERIALS AND METHODS

### Collection of plant material

The shoots of plant *Enicostemma littorale* Blume were collected from Karnataka (India) from August to September, 2005) at the end of flowering season and were authenticated By Prof. K. Prabhu, Department of Pharmacognosy, S. C. S. College of Pharmacy, Harapanahalli. The voucher specimen was deposited at SCSCOP, Harapanahalli.

### Preparation of the extract

The plant material was dried under shade. The shade-dried material was powdered. The coarse powder was subjected to successive extraction using solvents with increasing order of polarity that is, petroleum ether, chloroform, and methanol, and macerated with chloroform water.[8] All the extracts were subjected to the preliminary phytochemical tests and the methanolic extract showed the presence of flavonoids. Hence, the methanolic extract was selected for further studies.

### Animals

Albino rats (125 – 175 g) and mice (18 – 25 g) of either sex were obtained from NIMHANS, Bangalore, and were kept in standard plastic animal cages in a group of six to eight in each cage, at standard conditions, with 12 hours of light and dark cycle, in an institutional animal house. The animals were fed with the standard rodent diet and with water *ad libitum*. After one week of acclimatization the animals were used for further experiments. The CPCSEA approval number was Reg. No.157/1999/CPCSEA. Approval from the institutional animal ethical committee for the usage of animals in the experiments and experimental protocol was obtained as per the Indian CPCSEA guidelines for the usage of laboratory animals prior to the experimentation.

### Acute toxicity studies

The extracted methanol was tested for acute toxicity studies as per CPCSEA guideline No. 420. No animals died even at 2000 mg/kg and hence one-tenth and one-fifth of 2000 mg/kg was selected for further investigations.

### Anti ulcer activity

#### Aspirin (ASP)-induced ulcer[9]

Albino rats of either sex weighing between 180 and 200 g were divided into five groups of six animals each and fasted for 24 hours with water *ad libitum*, prior to the experiment. The animals of groups 1 and 2 were pre-treated with vehicle and the animals of group 3 were treated with standard, that is, lansoprazole 8 mg/kg. Similarly the animals of groups 4 and 5 were pre-treated with methanolic extract 200 mg/kg and 400 mg/kg, respectively. Aspirin (200 mg/kg p o) was administered to the animals of groups 2 – 5, 60 minutes after the respective treatments. The animals were then sacrificed by cervical dislocation after six hours. The stomach was taken out and cut open along the greater curvature. The number of ulcers per stomach were noted and severity of the ulcers were observed microscopically and scoring was done as follows:[10] Zero for normal colored stomach, 0.5 for red coloration, 1 for spot ulcer, 1.5 for hemorrhagic streaks, 2 for ulcer > 3 but < 5 mm, and 3 for ulcer > 5 mm. The mean ulcer score for each animal was expressed as the ulcer index. The percentage protection was calculated.

#### Ethanol induced (EtoH)-induced ulcer[11]

Albino rats of either sex weighing between 180 – 200 g were divided into five groups of six animals each and fasted for 24 hours with water *ad libitum*, prior to the experiment. The animals of groups 1 and 2 were pre-treated with vehicle and the animals of group 3 were treated with standard, that is, lansoprazole 8 mg/kg. Similarly the animals of groups 4 and 5 were pre-treated with methanolic extract 200 mg/kg and 400 mg/kg, respectively. Ethanol (100% 1 ml/200 g, p o) was administered to all the animals of groups 2 – 5, 60 minutes after the respective treatments. The animals were sacrificed by cervical dislocation after one hour of EtOH administration and the stomach was incised along the greater curvature and examined for ulcers. The ulcer index was scored as mentioned earlier, using Kulkarni’s method,[10] and the percentage protection was also reported.

#### Pylorus-ligated (PL) rats[12]

Albino rats of either sex weighing between 180 – 220 g were divided into five groups of six animals each, fasted for 18 hours and care was taken to avoid coprophagy. Control vehicle (group-2) or standard drug (group-3) or extracts (group - 4 and 5) were administered 60 minutes prior to Pyloric ligation, under light ether anesthesia. The abdomen was opened and pylorus ligation was done without causing any damage to its blood supply. The stomach was replaced carefully and the abdomen wall was closed in two layers with interrupted sutures. The animals were deprived of water during the post-operative period. After four hours, the stomach was dissected out, and the contents were collected into tubes for estimation of biochemical parameters. The stomach was taken out and cut open along the greater curvature and ulcers were scored and % protection was reported as mentioned in the above explained models.

### Gastric secretion

The gastric juice was collected four hours after pylorus ligation and centrifuged for five minutes at 2000 rpm and the volume of the supernatant was noted. The pH of the gastric juice was recorded by the pH meter. Then the contents were subjected to analysis for free and total acidity. Free acidity output was determined by treating with 0.01N NaOH, using the Topfer’s reagent until the solution turned yellowish orange. Then titration was continued, with phenolphthalein as an indicator, until there was a red tinge as the end point. This volume corresponded to total acidity.

### *In vitro* Anti-inflammatory activity

Inhibition of albumin denaturation was studied according to Muzustima and Kabagashi, with slight modifications[13] The test compounds were dissolved in a minimum amount of dimethyl formamide (DMF) and diluted with phosphate buffer (0.2 M, PH 7.4). The final concentration of DMF in all the solutions was less than 2.5%. The test solution (1 ml) containing 100 and 200 mg of the drug was mixed with 1 ml of 1 mM albumin solution in phosphate buffer and incubated at 27° ± 1° C for 15 minutes. Denaturation was induced by keeping the reaction mixture at 60° ± 1° C in a water bath for 10 minutes. After cooling the samples, the turbidity was measured. Percentage inhibition of denaturation was calculated from control. Each experiment was done in triplicate and the average was taken.

### Statistical analysis

All the results are subjected to Student’s T test and the ‘ *P* ’ value less than 0.05 was considered as statistically significant.

## RESULTS

The observations of the +ve control group indicated that aspirin (200 mg/kg)-induced gastric ulcers were to the extent of 17.10 ± 1.45 (ulcer index). Pre-treatment with test extracts reduced the ulceration in a dose-dependent manner. The extent of the gastroprotective effect of the test extracts was 63.28 and 78.14% at 200 mg/kg and 400 mg/kg doses, respectively, which was comparable to that of the standard lansoprazole 8 mg/kg. Similar results were obtained with the ethanol-induced ulcer model also. The test extract showed gastroprotection in a dose-dependent manner, that is, 61.54 and 76.9% protection at 200 and 400 mg/kg doses, respectively. The test extracts at the doses mentioned earlier showed a significantly higher level of protection than that of standard lansoprazole (8 mg/kg). The results are compiled in Table 1.

**Table 1:** Effect of Methanolic extract of leaves of *Enicostemma littorale* Blume on Aspirin, Ethanol (1 ml/kg), and four-hour Pylorus ligation (PL)- induced gastric ulcers in rats

Treatment	Dose	Ulcer index			% of protection		
		Aspirin	Ethanol	PL	Aspirin	Ethanol	PL
−ve Control	–	0.680 ± 0.056	0.333 ± 0.123	0.680 ± 0.056	-	-	-
+ve Control	–	17.10 ± 1.45	15.16 ± 2.18	9.16 ± 0.92	-	-	-
Lansoprazole	8 mg/kg BW	5.83 ± 1.40*	6.75 ± 0.91*	3.00 ± 0.51**	66.02	54.47	67.24
MeOH ext of EA	200 mg/kg BW	6.33 ± 0.67*	5.83 ± 0.55*	5.16 ± 0.69*	63.28	61.54	43.66
MeOH ext of EA	400 mg/kg BW	3.75 ± 0.61*	3.50 ± 0.28*	3.25 ± 0.61**	78.14	76.91	64.51

* < 0.01

* < 0.001 vs. Control, EA: *Enicostemma littorale* Blume

The pyloric ligation caused the accumulation of gastric secretions (7.3 ml) with pH 2.35. The total acidity and free acidity of the gastric secretions were 130.80 and 87.50, respectively. Pre-treatment with the test extracts reduced the volume of gastric secretion (5.13 and 3.01 ml at 200 and 400 mg/kg doses) and the pH was elevated up to 6.56. In addition the total acidity and free acidity were also reduced significantly in a dose-dependent manner. The results are compiled in Table 2. Furthermore, it was observed that pyloric ligation caused gastric ulcerations and pretreatment with test extracts reduced them significantly in a dose-dependent manner [Table 1]. In this model also the gastroprotection offered by the test extracts were comparable to those of the standard lansoprazole 8 mg/kg.

**Table 2:** Effect of Methanolic extract of leaves of *Enicostemma littorale* Blume on Gastric secretion following Pyloric Ligation-induced Ulcer in Rats

Treatment	Dose	Volume (ml)	pH	Total Acidity (Eq/I)	Free Acidity (Eq/I)
Control	--	7.30 ± 0.57	2.35 ± 0.16	130.80 ± 6.41	87.50 ± 2.32
Lansoprazole	8 mg/kg BW	1.68 ± 0.19**	7.38 ± 0.08**	42.33 ± 1.35**	34.83 ± 2.30**
MeOH ext of EA	200 mg/kg BW	5.13 ± 0.36*	3.61 ± 0.29*	76.00 ± 2.28**	64.5 ± 2.47**
MeOH ext of EA	400 mg/kg BW	3.01 ± 0.18*	6.56 ± 0.26**	56.66 ± 2.59**	42.50 ± 2.54**

* < 0.01

* < 0.001, EA: *Enicostemma littorale* Blume

## DISCUSSION

It is evident from the present study that the study extract is non-toxic even at 2000 mg/kg and possesses gastroprotective and anti-inflammatory activity against the experimental models.

The results are concurrent with the fact that plants containing antioxidant principles also possess antiulcer properties. As the antioxidant-containing extract demonstrates antiulcer and anti-inflammatory properties, it may be inferred that the free radicals may also have a role in causing ulcers and inflammation. This is supportive evidence for the reports in literature regarding this. Similarly it also justifies the traditional usage of this plant for gastrointestinal disturbances. However, the test extract also shows the anti-inflammatory effect with gastroprotection. This is contrary to the fact that anti-inflammatory agents cause gastric irritation and hyperacidity. This aspect has to be verified and probably different phytoconstituents may be responsible for both the beneficial effects of the study extract.

It has been proposed that in pyloric ligation, the digestive effect of accumulated gastric juice and hindrance of gastric blood circulation is responsible for the induction of ulceration.[14] The antiulcer activity of *Enicostemma littorale* in the pylorus ligation model is evident from its significant reduction in gastric volume, free acidity, total acidity and ulcer index. As *Enicostemma littorale*-treated animals significantly inhibit the formation of ulcers in pylorus-ligated rats and also decrease both concentration and gastric volume, it is suggested that *Enicostemma littorale* can suppress the gastric damage induced by aggressive factors.

Ethanol-induced gastric injury is associated with significant production of oxygen-free radicals leading to increased lipid peroxidation, which causes damage to the cell and the cell membranes.[15] *Enicostemma littorale* significantly protects the gastric mucosa against the ethanol challenge as shown by the reduced values of the ulcer index, as compared to the solvent control group, suggesting its potent gastroprotective effect, which may be due to its reported antioxidant activity [Table 3].[16]

**Table 3:** Inhibition of bovine serum albumin denaturation by methanolic extract of *Enicostemma littorale* Blume (EA)

Compound	Absorbance	Inhibition of denaturation (%)
Control	0.098 ± 0.009	–
MeOH ext of EA 100 mg	0.121 ± 0.002*	23.46
MeOH ext of EA 200 mg	0.135 ± 0.001*	37.75
MeOH ext of EA 300 mg	0.140 ± 0.001*	42.85
Standard diclofenac sodium (0.2mM)	0.167 ± 0.033*	70.40

* All compounds tested at 0.2 mm concen

Nonsteroidal anti-inflammatory drugs (NSAIDs) like aspirin, cause gastric mucosal damage by decreasing the prostaglandin levels through inhibition of PG synthesis.[17] *Enicostemma littorale* was significantly effective in the protective gastric mucosa against aspirin-induced ulcers at the entire dose level studied.

Hence, it may be inferred that *Enicostemma littorale* affords effective protection to the gastric mucosa against various insults, by increasing the gastric mucin content and decreasing the acid volume and free and total acidity in rats. The effects in all the three models studied were dose-dependent.

There is a report that the plant possesses flavonoids and flavonoids are known to have antioxidant activity. As there are reports that the generation of free radicals is one of the causes of the ulceration, the antiulcer activity of the extract may be attributed to the antioxidant potential of the plant.[16]

Medical treatment of peptic ulcers is dependent on correcting the imbalance between the offensive and defensive factors. The methanolic extract of the study plant acts on both parameters of the equation, which govern the treatment of peptic ulcer and thus can be useful clinically. Clinical usefulness of *Enicostemma littorale* is thus specially indicated in conditions such as rheumatoid arthritis, where the prostaglandin synthesis inhibitors are usually used with the concurrent danger of gastric erosion / ulcers. *Enicostemma littorale* may prove beneficial in arthritic conditions, with no danger of gastrointestinal distress. However, further experiments are required to establish and elaborate the molecular mechanisms of its antiulcer and anti-inflammatory activities.
